# Enhancing weed detection through knowledge distillation and attention mechanism

**DOI:** 10.3389/frobt.2025.1654074

**Published:** 2025-09-11

**Authors:** Ali El Alaoui, Hajar Mousannif

**Affiliations:** 1 Computer Science Department, Computer Systems Engineering Laboratory, Faculty of Sciences Semlalia Cadi Ayyad University, Marrakesh, Morocco; 2 LAMIGEP, Moroccan School of Engineering Sciences, Marrakech, Morocco

**Keywords:** deep learning, precision agriculture, vision transformer, weed detection, robotic weed control

## Abstract

Weeds pose a significant challenge in agriculture by competing with crops for essential resources, leading to reduced yields. To address this issue, researchers have increasingly adopted advanced machine learning techniques. Recently, Vision Transformers (ViT) have demonstrated remarkable success in various computer vision tasks, making their application to weed classification, detection, and segmentation more advantageous compared to traditional Convolutional Neural Networks (CNNs) due to their self-attention mechanism. However, the deployment of these models in agricultural robotics is hindered by resource limitations. Key challenges include high training costs, the absence of inductive biases, the extensive volume of data required for training, model size, and runtime memory constraints. This study proposes a knowledge distillation-based method for optimizing the ViT model. The approach aims to enhance the ViT model architecture while maintaining its performance for weed detection. To facilitate the training of the compacted ViT student model and enable parameter sharing and local receptive fields, knowledge was distilled from ResNet-50, which serves as the teacher model. Experimental results demonstrate significant enhancements and improvements in the student model, achieving a mean Average Precision (mAP) of 83.47%. Additionally, the model exhibits minimal computational expense, with only 5.7 million parameters. The proposed knowledge distillation framework successfully addresses the computational constraints associated with ViT deployment in agricultural robotics while preserving detection accuracy for weed detection applications.

## 1 Introduction

As the global population grows rapidly, the demand for food is projected to increase by 70% by 2050 (Caldera and Breyer, 2019). To achieve both high yield and top-quality crop production, enhancing production capacity in the agricultural sector becomes crucial. Researchers have been actively addressing various challenges within agriculture to develop intelligent and precise machine learning solutions. Precision farming leverages concepts from artificial intelligence (AI) and robotics to create targeted solutions that can be applied at the level of individual plants, rather than entire fields. In the realm of precision agriculture, automatic weeding plays a crucial role by identifying and targeting individual weeds. Deep learning has garnered significant interest for its effectiveness in detecting, classifying, and segmenting weeds. Various methodologies and approaches have demonstrated the efficacy of convolutional neural network-based methods in addressing vision-related tasks, such as object detection, object classification, and object segmentation, etc. The recent advancements in neural networks reveal that attention-based transformer modules can serve as a complete replacement for convolutional operations. Additionally, researchers have explored joint designs that combine both attention-based transformers and convolutions, aiming to foster symbiosis between these two complementary sets of operations (Vaswani et al., 2017). Within the domain of deep learning, Transformers have demonstrated significant achievements in natural language processing. However, their application to computer vision was previously limited. The emergence of Vision Transformers (ViT) revolutionized this landscape by directly employing the Transformer architecture on image patches, resulting in exceptional performance for image classification tasks (Dosovitskiy et al., 2021). Nevertheless, numerous research papers addressing weed detection, classification, or segmentation employ Convolutional Neural Network (CNN) architectures. Weed detection in soybean crops has been explored using Convolutional Neural Networks (CNNs) by dos Santos Ferreira et al. (2017). Espejo-Garcia et al. (2020a) explored the potential of utilizing transfer learning techniques for detecting two weed species. They employed five pre-trained convolutional networks (Xception, Inception-Resnet, VGNets, Mobilenet, and Densenet). Additionally, they adopted a comprehensive real-time weed detection strategy based on a cascade classifier trained with Haar-like features. Saleem et al. introduced an innovative methodology grounded in deep learning (DL) for weed detection in (Saleem et al., 2022). This approach encompasses the utilization of diverse neural network architectures, both single-stage and dual-stage, and examines the impact of assorted image resizing methodologies. Ali et al. in (El Alaoui et al., 2022), address the challenges of weed variation and complex agricultural backgrounds by employing a data fusion approach. Their work highlights the potential of CNNs (YoloV5) for accurate weed identification and targeted weed control. To emphasize the recent trends in deep learning, Hasan et al. conducted an exhaustive review of the literature on weed detection and classification methods within the context of deep learning (DL). Despite considering only 70 articles, they rigorously evaluated each one using consistent criteria. Additionally, the authors delineated shared concepts for the application of DL in agriculture (Hasan et al., 2021). Although this architecture has been successful in overcoming certain obstacles, CNNs continue to encounter numerous difficulties, including substantial computational demands, the need for global context understanding, inherent inductive biases, and comprehensive feature representation, among others.

Nevertheless, a limited number of studies have employed Vision Transformers to tackle this challenge. Same way Reenul et al., have embraced the self-attention capabilities of Vision Transformer (ViT) models for the classification of various plant species, such as red beet, off-type beet with green leaves, parsley, and spinach. Their empirical studies demonstrate that ViT models, even when trained on a small dataset of labeled examples, outperform conventional state-of-the-art CNN-based models like EfficientNet and ResNet. The ViT models achieved an impressive top accuracy of 99.8% (Reedha et al.). Similarly in (Jiang et al., 2022), They conducted a comprehensive evaluation of three distinct Transformer architectures Swin Transformer, SegFormer, and Segmenter, specifically for the task of weed segmentation. Their findings revealed that the SegFormer model attained a notable Mean Accuracy (mAcc) of 75.18% and a Mean Intersection over Union (mIoU) of 65.74%. A novel lightweight Vision Transformer approach for weed mapping from high-resolution drone imagery, achieving superior segmentation and enabling efficient herbicide management through innovative transfer learning techniques has been proposed by Castellano et al. (2023).

Resource limitations and training costs pose challenges for deploying deep learning models for weed detection. This study tackles these limitations by proposing a knowledge distillation method to optimize the ViT model. The approach aims to reduce training costs, data requirements, and model size while maintaining weed detection performance.

In summary, the contributions of this paper can be delineated as follows:

- Novel CNN-to-ViT Knowledge Distillation Framework: We introduce a computationally efficient knowledge distillation approach that enables effective transfer of inductive biases, parameter sharing mechanisms, and local feature extraction capabilities from a CNN-based teacher model (ResNet-50) to a lightweight Vision Transformer student architecture, while preserving the self-attention mechanisms for global feature modeling in agricultural weed detection tasks.
- High-Performance Lightweight Model for Edge Deployment: Our compact ViT-based student model achieves superior weed detection accuracy (83.47% mAP) with minimal computational overhead (5.7M parameters), making it readily deployable on resource-constrained edge devices for real-time precision farming applications while outperforming existing state-of-the-art methods.
- Rigorous Real-World Validation: The proposed framework is validated on an authentic agricultural dataset collected under field conditions with manual annotations, demonstrating high ecological validity and practical applicability for real-world deployment scenarios in precision agriculture.

This paper is organized into four sections. It opens with an introduction and a review of related works. Next, the proposed method and materials are detailed. This is followed by a discussion of the experimental results, highlighting key findings. The paper concludes with a summary of the contributions and implications of the study.

## 2 Related work

### 2.1 Object detection

Object detection has emerged as a highly favored task in the field of computer vision, owing to its wide-ranging applications that address numerous practical issues. It involves the process of identifying and locating one or several objects within an image, determining their categories, and pinpointing their positions. In recent years, Transformers have demonstrated remarkable efficacy in executing tasks related to object detection. In (Carion et al., 2020) introduced DETR (Detection Transformer), an innovative approach for object detection. This method is distinctive in its ability to directly predict collections of output elements, which include bounding boxes, class labels, and confidence scores. Notably, it accomplishes this without the necessity for distinct stages of region proposal and subsequent refinement unlike CNN-based models. Michael et al. (Yang, 2025) showcased that the DetTransNet model markedly improves object detection on the COCO dataset, achieving this feat without an increase in parameter count. The model notably outperforms established baselines, registering a 1.2% uplift in Average Precision. Baseline models. In the context of transformer-based models, a critical factor influencing their effectiveness lies in the pre-training phase. Previous research, as highlighted by the study referenced (Dosovitskiy et al., 2021), emphasizes the requirement for a large volume of meticulously curated data during pre-training. DeiT (Touvron et al., 2021) employs innovative training strategies that allow it to perform well even with limited data. These policies contribute to its efficiency, making it a viable option for scenarios where large-scale datasets are not available.

### 2.2 Weed detection

Machine learning (ML) has demonstrated significant effectiveness in developing automatic weed detection and classification systems. These models can be deployed across a wide range of applications. In this part, we offer a concise overview of research previously conducted in this context. Deep Learning (DL) enables machines to autonomously identify the most distinctive attributes of objects within unprocessed images. Compared to conventional Machine Learning (ML) approaches, DL exhibits greater resilience to diverse alterations in the input images, which contributes to enhanced outcomes in Detection, classification and segmentation tasks. Espejo-Garcia et al. (2020) developed a crop/weed identification system combining fine-tuned deep learning models with traditional classifiers, achieving high accuracy and avoiding overfitting. Utilizing a diverse dataset from Greece, the best model, a fine-tuned Densenet with SVM, reached a 99.29% F1 score, demonstrating robust performance across different conditions. Rai et al. (2024) have developed the YOLO-Spot model, a streamlined version of the YOLOv7-tiny framework, designed for efficient weed identification within agricultural settings. The model variant, YOLO-Spot_M, stands out for its enhanced precision and lower energy requirements, rendering it an ideal candidate for incorporation with remote sensing technologies to facilitate accurate weed control. Olsen et al. (2019). Have created DeepWeeds, the first extensive, public image dataset for Australian weed species, enabling the advancement of classification methods for automated weed control. The dataset includes 17,509 images of eight significant weeds, with deep learning models Inception-v3 and ResNet-50 achieving classification accuracies of 95.1% and 95.7% respectively, and showcasing real-time inference capabilities. Convolutional Neural Networks (CNNs) adeptly apply convolutional filters to parse images, extracting pivotal features crucial for object identification. This process is underpinned by the network’s inherent mechanisms: local receptive fields, shared weights, and translational equivariance, which collectively enhance the network’s visual interpretative capabilities. The majority of studies concentrating on the classification, identification, or segmentation of weeds utilize CNNs-based models structures (Dian Bah et al., 2018; Suh et al., 2018; Nkemelu et al., 2018) such as Inception-v3 (Szegedy et al., 2015), GoogLeNet (Kerkech et al., 2019), ResNet-50, ResNet-101 (He et al., 2015), AlexNet (Krizhevsky et al., 2012), VGG-16, VGG-19 (Simonyan and Zisserman, 2014).

The vision transformer (ViT) signifies a transformative advance in employing attention models for computer vision tasks, owing to the benefits provided by the attention paradigm, While their application to address agricultural tasks is limited, only a few studies have utilized ViTs for weed detection due to the scarcity of data. The GNViT model in (P and I. M, 2024), utilizing a pre-trained vision transformer (ViT) on the ImageNet dataset, aims to detect and classify pests affecting groundnut crops. Rigorous evaluation using IP102 dataset revealed GNViT’s superior accuracy (99.52%) compared to state of the art models. These findings highlight the potential of ViTs like GNViT in enhancing crop security and reducing losses. Nevertheless, according to this study (Rozendo et al., 2024), the PVT (Pyramid Vision Transformers) models for weed classification, highlighting that an ensemble of these methods can achieve up to 99.17% accuracy with minimal training. The results underscore the potential of these models to significantly enhance weed detection in agriculture. Liang et al. (2021) use a ViT deep neural network for classifying soybean and weed images, demonstrating superior classification and generalization capabilities. The network, designed with specific parameters, effectively utilizes self-attention mechanisms for image semantic recognition.

#### 2.2.1 Vision transformer

The Transformer architecture, introduced by Vaswani et al. (2017), has become the benchmark model for all natural language processing (NLP) tasks. Initially designed for machine translation, it now serves as the reference standard across the field. ViTs have become a significant asset in computer vision (Dosovitskiy et al., 2021), utilizing self-attention mechanisms to analyze image data. In contrast to traditional convolutional neural networks (CNNs), ViTs excel at capturing global context, making them ideal for complex tasks. Recent research has highlighted the effectiveness of ViTs in diverse agricultural applications, including crop disease detection, yield prediction, and precision farming, underscoring their versatility and potential to advance agricultural technology.

The input goes through six stages of a vision transformer to obtain the class id:

1. Image Patching: The input image is divided into a grid of fixed-size ( 16 ∗ 16) patches. Each patch is treated as a token, similar to words in natural language processing (NLP).
2. Linear Embedding: Each image patch is flattened into a 1D vector and then linearly transformed into a lower-dimensional embedding. This step converts the spatial information of the patches into a format suitable for the transformer.
3. Positional Encoding: Since transformers do not inherently understand the order of tokens, positional encodings are added to the patch embeddings. These encodings provide information about the position of each patch within the original image.
4. Transformer Encoder: The sequence of patch embeddings, now with positional encodings, is fed into a standard transformer encoder. The encoder consists of multiple layers of self-attention and feed-forward neural networks. This stage allows the model to capture complex relationships between different patches.
5. Classification Token: A special classification token is prepended to the sequence of patch embeddings. This token aggregates information from all patches and is used for the final classification task.
6. Output Layer: The output from the transformer encoder is passed through a small multi-layer perceptron (MLP) head. The classification token’s output is used to produce the final prediction.

#### 2.2.2 Knowledge distillation

Knowledge Distillation (KD) (Hinton et al., 2015) leverages the teacher model’s output as soft labels to guide the student model, resulting in significant enhancements for lightweight models without additional inference costs. This technique has been extensively investigated for Convolutional Neural Network (CNN) models and has been effectively applied to various vision tasks, including object detection, object classification and object segmentation.

The objective is to minimize the Kullback-Leibler divergence between the softmax outputs of the teacher and student models. Let $Z_{t}$ represent the logits of the teacher model and $Z_{s}$ the logits of the student model. We denote the temperature for distillation by τ , the coefficient balancing the Kullback-Leibler divergence loss (KL) and the cross-entropy loss (LCE) on ground truth labels y by α , and the softmax function by σ (Wei et al., 2020). The distillation objective is:

$$\begin{array}{ll} L_{\text{distill}} & =\alpha \cdot \text{KL}\left(\sigma \left(Z_{t}/\tau \right),\sigma \left(Z_{s}/\tau \right)\right)+\left(1-\alpha \right)\cdot \text{LCE}\left(y,\sigma \left(Z_{s}\right)\right) \end{array}$$

## 3 Methods and materials

This paper contributes to the application of machine vision technologies for weed control in real agricultural environments. During the deployment stage, both model performance and model size are critical considerations. A prevalent issue is the trade-off between accuracy and model size. Highly accurate deep learning models typically possess a large number of parameters, rendering their deployment expensive or even unfeasible. In this section, we introduce a novel architecture designed to address this challenge. We employ a knowledge distillation technique that incorporates an attention mechanism, enabling us to reduce the model size while preserving its accuracy. Furthermore, This leverages advantages such as parameter sharing and local feature extraction from a large pre-trained CNN-model (ResNet-50) as a teacher for our small and straightforward ViT model. This approach enables more powerful, lightweight, and cost-efficient model for weed detection.

### 3.1 Model overview

Our knowledge distillation framework transfers learning from a ResNet50 teacher to a compact ViT student for efficient weed detection. The teacher extracts hierarchical features through convolutional layers, while the student processes 16 × 16 image patches via transformer blocks with multi-head attention. Using a combined cross-entropy and distillation loss with temperature control, the system maintains accuracy while reducing parameters by 77.74% (to 5.7M). This integrates CNN’s local feature extraction with ViT’s global attention, enhanced by patch augmentation for robust field performance in precision agriculture.

**FIGURE 1:**
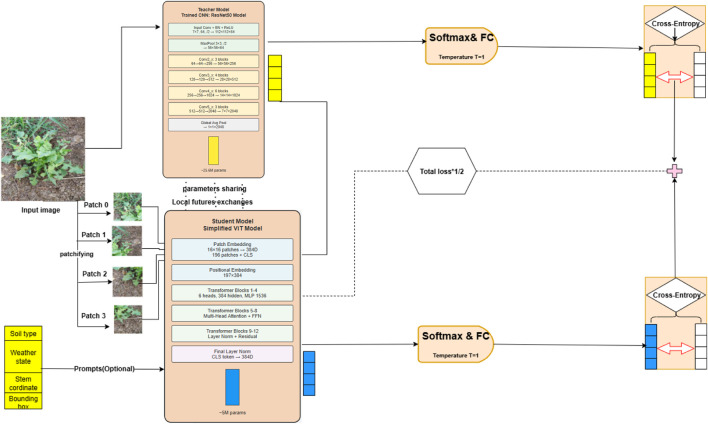
Proposed architecture framework.

**FIGURE 2:**
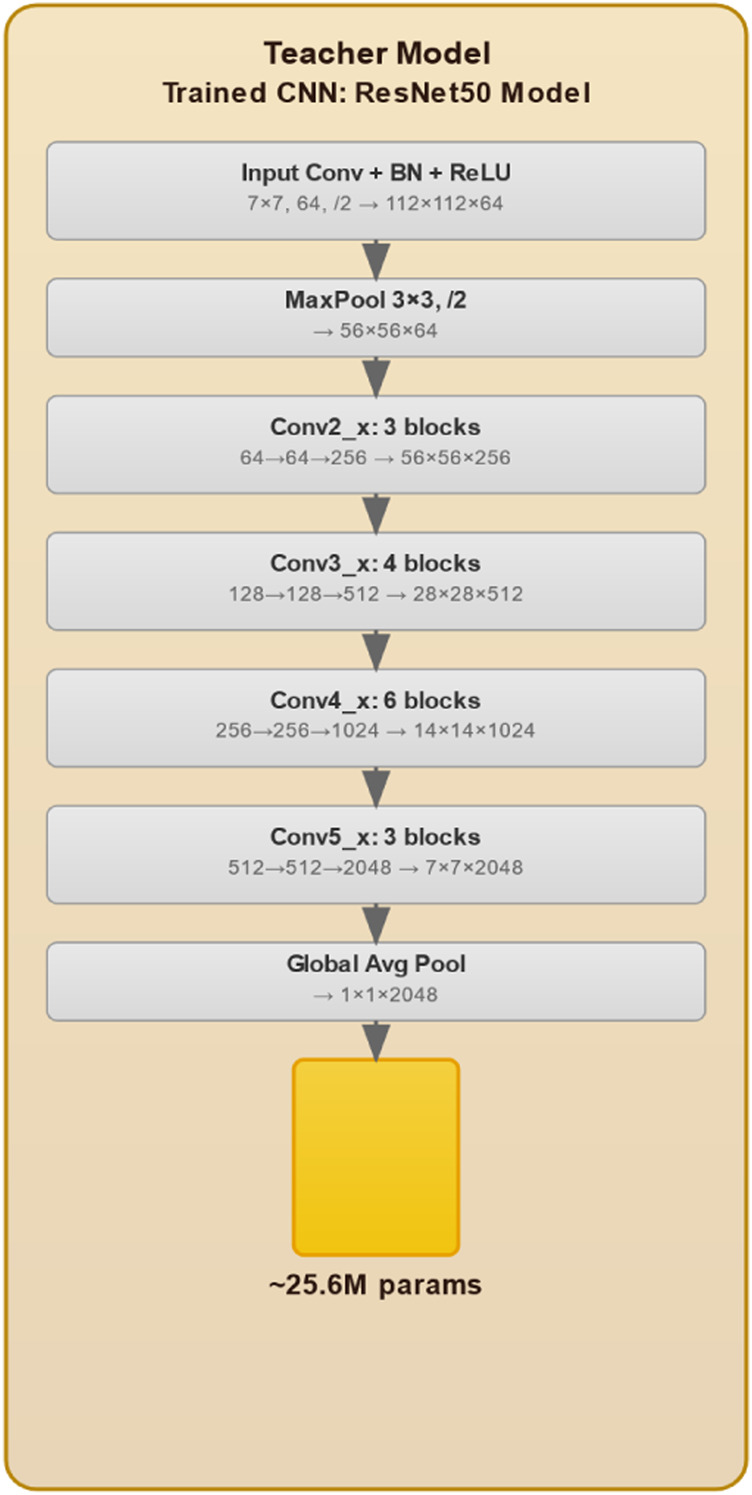
Teacher model architecture specifications.

**FIGURE 3:**
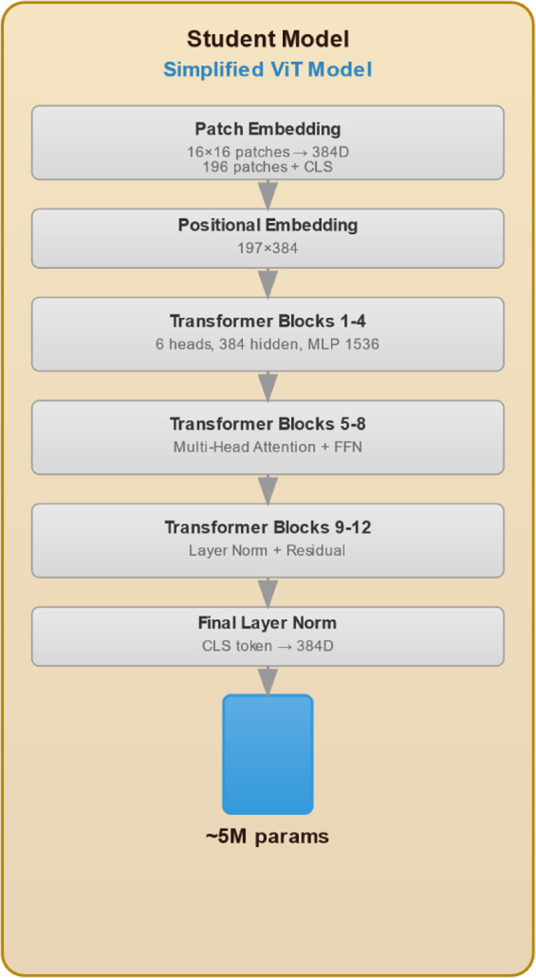
Student model architectural design.

The proposed knowledge distillation framework for weed detection, illustrated in Figure 1, encompasses an advanced three-phase methodology that systematically transfers knowledge from a robust ResNet50 teacher network to an efficient ViT student model. This approach addresses the critical challenge of deploying computationally intensive deep learning models in resource constrained agricultural environments while maintaining high detection accuracy. The initial phase involves comprehensive training of the ResNet50 teacher model (see Figure 2), which processes input images through a hierarchical CNN architecture. The model begins with a 7 × seven convolutional layer followed by batch normalization and ReLU activation, progressively down sampling the spatial dimensions through four residual stages to extract high level features. The final global average pooling layer condenses these features into a 2048 dimensional representation, leveraging 25.6M parameters to capture intricate visual patterns in agricultural imagery. The second phase constitutes the core innovation: systematic knowledge transfer from the teacher to the student ViT model (see Figure 3), which adopts a lightweight transformer based design. The student processes input images by splitting them into 16 × 16 patches, linearly embedding each into 384 dimensional vectors (totaling 196 patches plus a CLS token). These embeddings are combined with positional information and processed through 12 transformer blocks. The blocks employ multi-head attention (6 heads) and feed forward networks (MLP size 1536), with layer normalization and residual connections ensuring stable training. Despite its streamlined architecture (5.7M parameters), the ViT student effectively mimics the teacher’s feature extraction capabilities through refined soft distillation. The knowledge distillation process employs a carefully designed loss function combining traditional cross entropy loss with a distillation loss component, where the temperature parameter fine tunes probability distribution softness. The student’s training further benefits from patch based data augmentation, enhancing its ability to generalize across spatial contexts. The final phase validates the distilled student model, demonstrating successful integration of CNN-derived hierarchical features with the ViT’s global self-attention mechanisms. The student’s architecture culminating in a layer-normalized CLS token classification proves particularly adept at modeling long-range dependencies in weed detection tasks. Experimental results confirm that the distilled ViT not only retains the teacher’s accuracy but does so with 77.74% fewer parameters, making it ideal for deployment on edge devices in precision farming.

### 3.2 Teacher model architecture

In our knowledge distillation process (He et al., 2015), we employ a pre-trained ResNet-50 model (On our dataset) as the teacher architecture. ResNet-50, consisting of 50 layers, utilizes a residual learning approach to facilitate the learning of complex features and mitigate the degradation problem. The final fully connected layer is adapted to output two neurons, corresponding to the weed and crop classes in our dataset. The architecture comprises multiple Residual Blocks, each containing two convolutional layers with a shortcut connection that enables direct information transfer to subsequent layers. This design aids in retaining information from earlier layers, thereby enhancing the model’s capacity to learn superior representations. Furthermore, global average pooling is applied before the fully connected layer to reduce the spatial dimensions of the feature maps. This is followed by a detection module composed of multiple feed-forward layers, tasked with predicting bounding boxes and class labels for the two specified target classes (See Figure 2).

Given the critical importance of small feature detection and overfitting prevention in weed detection tasks, ResNet-50 was chosen as the teacher network for its superior capabilities in these areas. Intricate morphological learning to differentiate weeds from crops across growth stages is made possible by its residual connections, which also address vanishing gradients. Through skip connections and multi-scale processing, the hierarchical architecture captures high-level semantic representations as well as fine-grained features (leaf textures, edge patterns). The residual design is appropriate for small agricultural datasets with high intra-class variability because it implicitly regularizes through identity mapping and ensemble effects. When paired with ImageNet pre-training, ResNet’s demonstrated ability to handle complex backgrounds, variable lighting, and species similarities makes it the best choice for knowledge transfer. The compact ViT student is guided to attain high performance with computational efficiency by the rich multi-level features of ResNet-50, which act as comprehensive supervisory signals. 83.47% mAP was attained during experimental validation, indicating practical viability for agricultural applications with limited resources.

### 3.3 Student model architecture

We employ a tiny ViT as the backbone for our student model, specifically designed to detect two classes: weed and crop. The tiny ViT model, trained on our dataset, leverages its efficient feature extraction capabilities. Our model architecture comprises three heads and 12 layers, with approximately 5.7 million parameters (See Figure 3). It operates with a training throughput of 54 frames per second (FPS), a dimension of 192, and a resolution of 224 × 224 pixels. To adapt tiny ViT for object detection, we integrate a detection head consisting of a series of feed-forward layers responsible for predicting bounding boxes and class labels for the two target classes. The combined architecture is trained end-to-end, optimizing both classification and localization tasks. Furthermore, For bounding box classification, we employ cross-entropy loss (Mao et al., 2023) alongside IoU loss (Zhou et al., 2019). The Softmax function (Franke and Degen, 2023) is used in the output layer to convert the network’s output into a probability distribution over the predicted classes. Additionally, we have adopted KL-divergence as the distillation loss function.

### 3.4 Advancements

To enhance the settings for advanced weed detection, we propose incorporating a Prompts List that includes parameters such as soil type, lighting conditions, and stem coordinates (Stem X and Stem Y). This addition aims to augment the performance of our student model by providing comprehensive contextual information. The student model leverages these additional prompts in conjunction with the guidance from the teacher model, ResNet50, which has been trained on a combination of the CottonWeedID15 and semi-Moroccan datasets. This integrated methodology capitalizes on both the detailed environmental prompts and the robust training of the teacher model, thereby will facilitating more precise and reliable weed detection.

#### 3.4.1 Data selection

**FIGURE 4:**
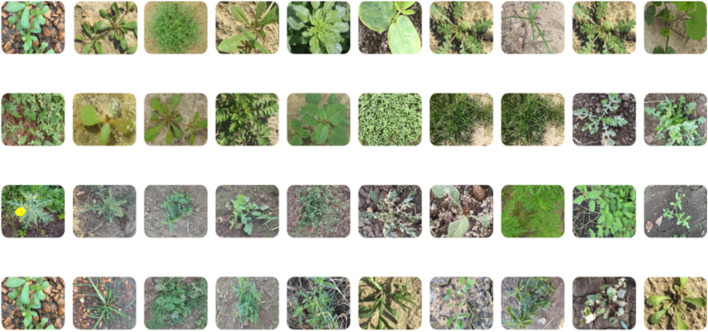
Sample images from the weed dataset used in this study.

To address the challenges presented by harsh environments, we established a comprehensive set of standards and criteria for dataset selection, taking into account various field-specific factors. These factors encompass the weed life cycle, scene background, natural soil conditions, occlusion, morphological variations in weeds, weed categories, weed coloration, geographical and seasonal plant variations, and fluctuating lighting conditions as illustrated in Figure 4. Following the application of a fusion technique (El Alaoui et al., 2022), the CottonWeedID15 and semi-Moroccan datasets were selected for this study as they comprehensively embody these characteristics as demonstrated in Table 1.

**TABLE 1:** Data selection criteria.

Norms	Conditions
Weather conditions	To create a dynamic dataset, most sample images need to be taken in a variety of weather conditions, including sunny, rainy, windy, cloudy and snowy
Seasonal variation Vs. weed change	The dataset comprises images collected throughout various times of the year, demonstrating the progressive morphological changes in weeds
Lighting conditions	To address the challenges posed by the demanding environment, we selected sample images captured under various lighting conditions, including darkness, sunlight, and plant shadows
The weed life cycle	The selected datasets should encompass various periods of seasonal weed growth
Scene background	All images should represent a diverse array of scenes and backgrounds, including sand, clay, silt, peat, chalk, and loam

Given the importance of these characteristics, our study achieves superior weed detection precision on the selected dataset, thereby demonstrating the robustness of our proposed approach. The dataset comprises approximately 6,323 images, including 5,187 RGB images from the CottonWeedID15 dataset. These images were captured in 2020 and 2021 using smartphones or handheld digital cameras under natural field illumination and at various stages of weed growth. Additionally, the semi-Moroccan dataset contributes 1,300 images, each a 512 × 512 color image captured under diverse field conditions. The dataset encompasses more than twenty classes of weeds, including one class of sesame crops considered as a negative class. We removed 160 images from our dataset because their characteristics did not meet our criteria, as shown in Table 1.

The dataset captures diverse weed species under real-world conditions, illustrating variations in morphology, background, soil texture, occlusion, and lighting. Selected from CottonWeedID15 and a Moroccan-contributed set, these samples reflect key criteria such as weed life cycle, coloration, and seasonal or geographic diversity ensuring a robust foundation for model training in complex agricultural environments.

### 3.5 Data-augmentation

Notably, One significant challenge in using transformers for certain vision tasks, particularly object detection, is their requirement for a larger dataset for training compared to convolutional models. To mitigate this issue, we leverage several data augmentation tactics such as Random Erasing (Wei et al., 2020), (Touvron et al., 2021), Rand-Augment (Cubuk et al.) and Auto-Augment (Dogus Cubuk et al., 2018). The experiments highlight the advantages of these methods and validate their efficacy in improving the student model’s performance, as measured by Mean Average Precision, especially when employing Rand-Augment over Auto-Augment.

## 4 Experiments

This section details a series of analytical experiments and the corresponding results derived from the proposed methodology. Initially, we discuss and analyze the outcomes obtained from the pre-trained ResNet50 model (teacher model), which was trained on the blend of the CottonWeedID15 and semi-Moroccan datasets (Chen et al., 2022), (El Alaoui et al., 2022). Subsequently,We fine-tune our simplified Vision Transformer (ViT) model (student model) on our dataset, both with and without the guidance of the teacher model, and assess its performance. The evaluation focuses on three critical metrics: mean average precision, frames per second (FPS), and model size.

**FIGURE 5:**
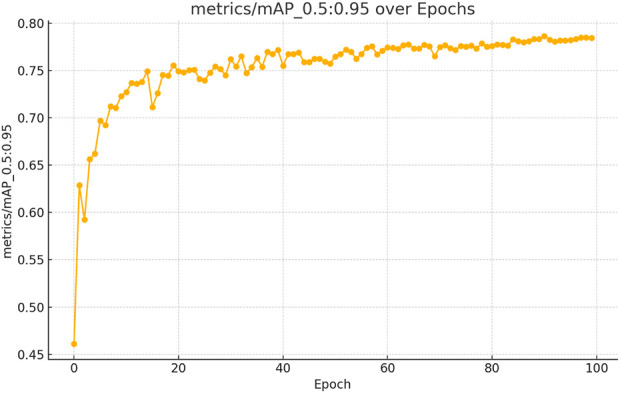
mAP convergence curve for the teacher model in weed detection tasks, computed across 100 epochs at IoU thresholds 0.5–0.95.

**FIGURE 6:**
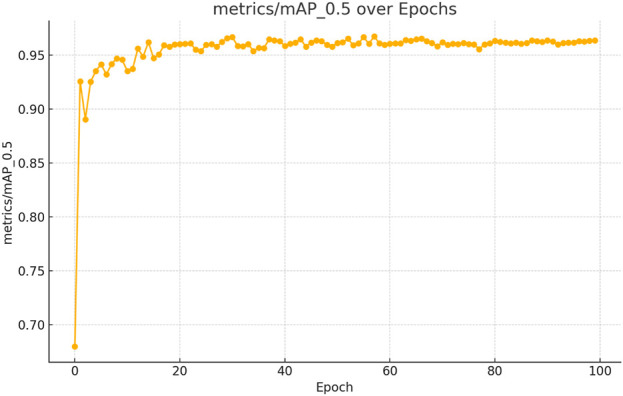
mAP convergence curve for the teacher model in weed detection tasks, computed across 100 epochs at IoU threshold 0.5.

**FIGURE 7:**
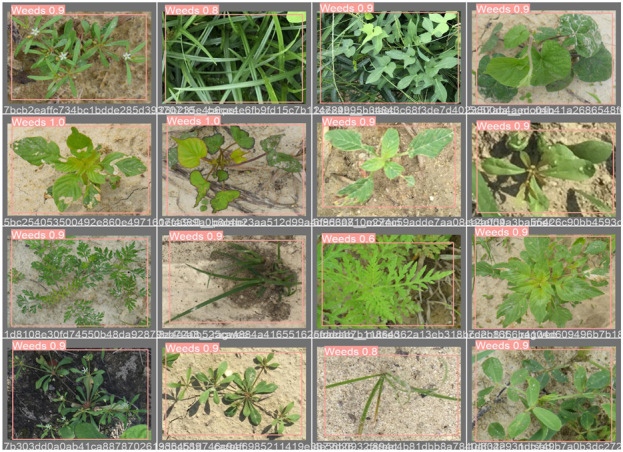
Performance evaluation of the teacher model on validation data.

**FIGURE 8:**
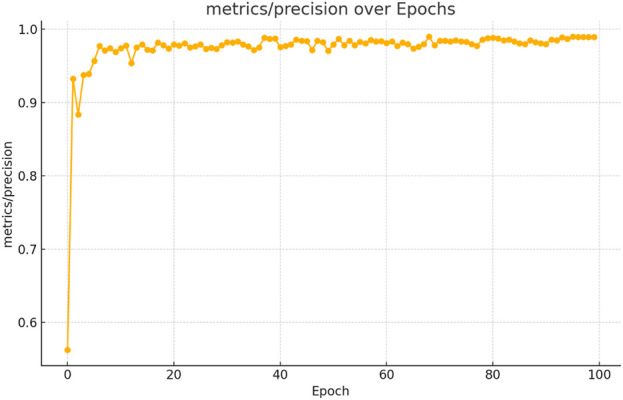
Teacher model precision performance progression in weed detection across 100 training epochs.

**FIGURE 9:**
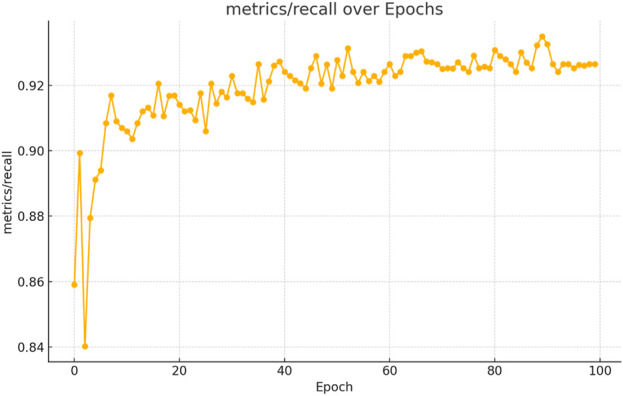
Teacher model recall performance progression in weed detection across 100 training epochs.

The figures above demonstrate the performance of our teacher model throughout the training phase on the complete dataset. The teacher model, as illustrated in Figures 5, 6, achieved a mean Average Precision (mAP) of 96.38% at an Intersection over Union (IoU) threshold of 0.5. This indicates a high level of accuracy in weed detection tasks (See Figure 7). The model’s precision, which measures the proportion of true positive detections among all positive detections, was 97.95% (See Figure 8). This high precision reflects the model’s ability to minimize false positives. Additionally, the recall rate, which measures the proportion of true positive detections among all actual positives, was 93.25% (See Figures 8, 9), indicating a strong ability to identify relevant objects. These results were obtained over 100 training epochs, demonstrating the model’s robustness and effectiveness in learning from the dataset.

**FIGURE 10:**
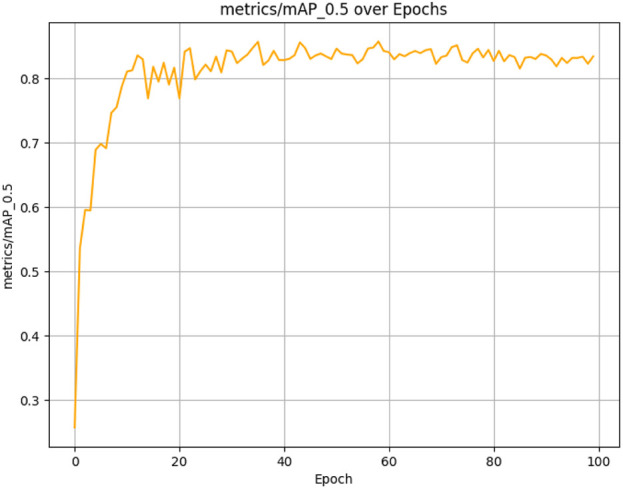
Student model mAP performance progression across 100 training epochs, assessed at IoU threshold of 0.5.

**FIGURE 11:**
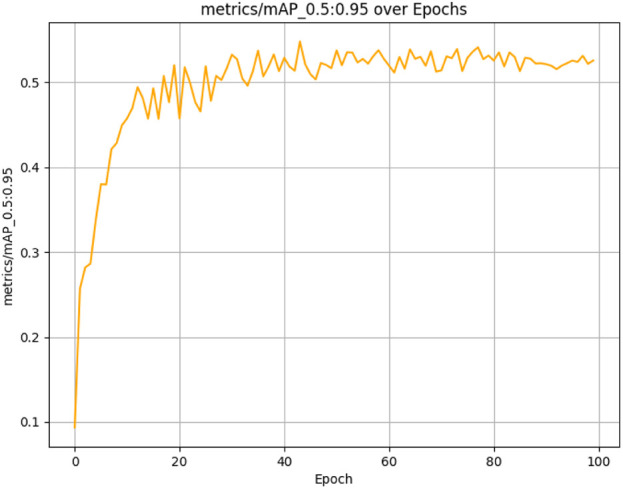
Student model mAP performance convergence across 100 training epochs evaluated at IoU thresholds 0.5–0.95.

**FIGURE 12:**
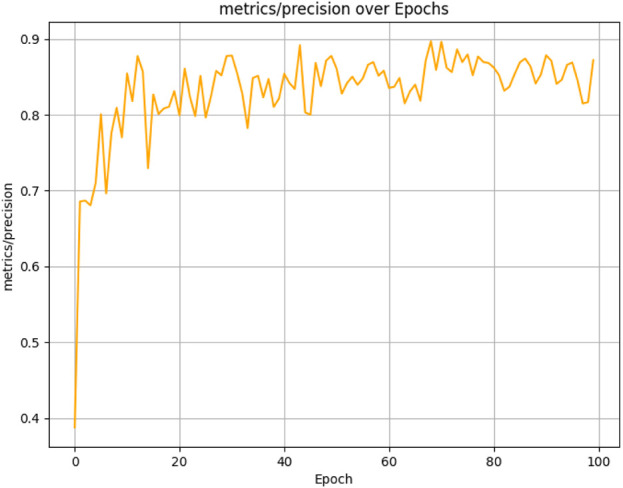
Student model precision performance progression in weed detection across 100 training epochs.

**FIGURE 13:**
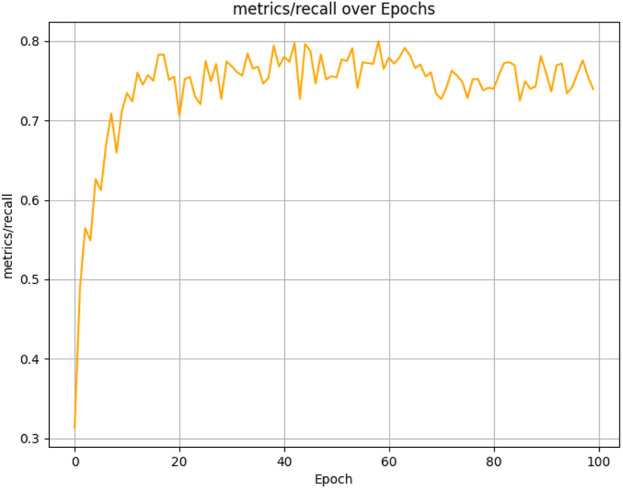
Student model recall performance progression in weed detection across 100 training epochs.

The results presented below illustrate the performance of the student model, which was evaluated over 100 epochs on our dataset using several critical metrics. The model achieved a mean Average Precision (mAP) of 83.47% at an Intersection over Union (IoU) threshold of 0.5 (See Figure 10) and a mean Average Precision (mAP) of 54.80% at an Intersection over Union (IoU) threshold of 0.5 0.95 (See Figure 11). Additionally, it demonstrated a Precision of 87.21% (See Figure 12) and a Recall of 73.93% (See Figure 13).

**FIGURE 14:**
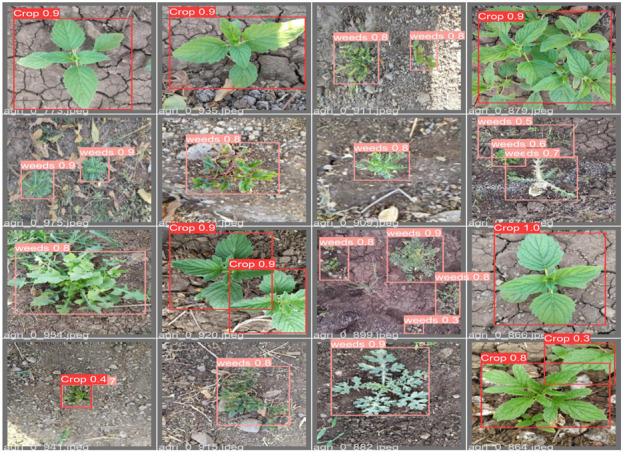
Performance evaluation of the student model on validation data.

These results, as demonstrated in Figure 14, show that the student model, guided by the ResNet-50 mentor model, achieves high accuracy in weed detection tasks. The elevated Precision value indicates a low rate of false positives, while the substantial Recall value reflects a strong ability to identify relevant objects within the dataset. Collectively, these metrics highlight the effectiveness of the mentor-student training approach in enhancing the performance of the student model.

We conducted a comprehensive ablation study comparing ResNet50, InceptionV3, and VGG19-based teacher networks on our weed detection dataset to determine the optimal backbone architecture. The ResNet50-based teacher achieved superior performance with 96.38% mAP@0.5, 97.95% precision, and 93.25% recall, outperforming VGG19-based teacher (94.12% mAP@0.5) and InceptionV3-based teacher (93.5% mAP@0.5). The residual connections in ResNet50 enable effective gradient flow and feature discrimination crucial for distinguishing subtle morphological differences between crops and weeds. Additionally, the ResNet50-based teacher demonstrates optimal computational efficiency with 45 FPS and 25.6M parameters compared to VGG19-based teacher’s 38 FPS with 32.4M parameters and InceptionV3-based teacher’s 30 FPS with 28.5M parameters. Based on this comprehensive evaluation, ResNet50-based architecture was selected as the teacher backbone due to its superior accuracy, computational efficiency, and effective knowledge transfer capabilities for weed detection tasks. The detailed performance comparison is presented in Table 1, demonstrating the ResNet50-based teacher’s consistent superiority across all evaluation metrics.

**FIGURE 15:**
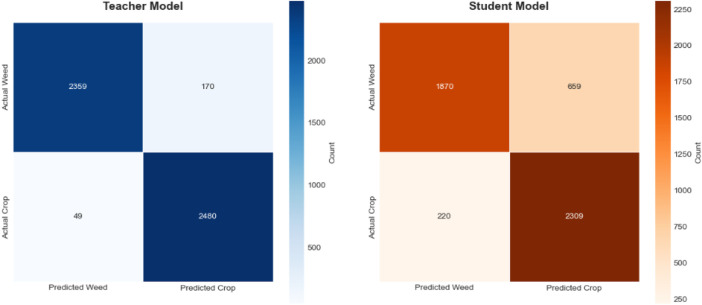
Comparative confusion matrices of teacher and student models for automated weed detection tasks on the experimental dataset.

**FIGURE 16:**
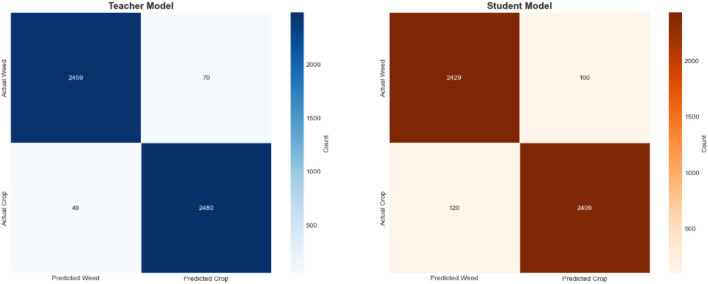
Confusion matrices comparing teacher and student model performance on weed detection tasks following Grad-CAM (Selvaraju et al., 2016) guided knowledge distillation implementation on the experimental dataset.

Our experimental results demonstrate that student model performance can be significantly enhanced through the integration of our proposed approach with Grad-CAM methodology. This combination facilitates more effective knowledge transfer from the teacher model to the student model by leveraging visual attention mechanisms that guide the distillation process. The Grad-CAM integration provides interpretable feature maps that enable the student model to better understand and replicate the teacher’s decision-making patterns, resulting in more robust feature representation learning, which subsequently improves weed detection accuracy (See Figures 15, 16).

The experimental results, as shown in Table 2, underscore the efficacy of the proposed method, which surpasses contemporary state-of-the-art techniques. This approach reduces the number of parameters by 77.74% compared to the teacher model, while preserving robust performance in weed detection, as evidenced by mAP and FPS metrics. The substantial parameter reduction demonstrates the model’s computational efficiency without compromising detection accuracy, making it particularly suitable for resource-constrained agricultural environments. Furthermore, the maintained performance metrics validate the effectiveness of our knowledge distillation framework in transferring critical feature representations from the complex teacher network to the streamlined student architecture. These findings support the adoption of vision transformers through knowledge distillation as lightweight models, characterized by low training and deployment costs on edge devices, thereby contributing to enhanced crop yields.

**TABLE 2:** Summary of the obtained results.

Metrics/Backbone + FPN	ResNet50	InceptionV3	VGG19	Student model
mAP@0.5	96.38%	93.5%	94.12%	83.47%
mAP@0.5:0.95	78.62%	75%	75.38%	52.56%
Precision	97.95%	92.40%	96.47%	87.21%
Recall	93.25%	90%	91.83%	73.93%
FPS(NVIDIA V100/A100)	45	30	38	54
Params	25.6M	28.5M	32.4M	5.7M

### 4.1 Implementation details

Our hyper-parameter settings and training strategy are as follows: To train both the student and teacher model architectures, the dataset was partitioned following a standard 80%-10%-10% split protocol. The training set comprised 80% of the total dataset (approximately 5,058 images), which was utilized to optimize the model parameters. A validation set consisting of 10% of the data was employed to monitor training progress, prevent overfitting, and mitigate gradient-related issues such as exploding or vanishing gradients. The remaining 10% of the dataset (633 images) was reserved as an independent test set to evaluate model performance on previously unseen samples, ensuring an unbiased assessment of the models’ generalization capabilities. For bounding box classification, we utilize cross-entropy loss (Mao et al., 2023) in conjunction with IoU loss (Zhou et al., 2019). The Softmax function (Franke and Degen, 2023) is adopted as the output layer to transform the network’s output into a probability distribution over the predicted classes. Moreover, we have adopted KL-divergence as the distillation loss function, facilitating the capture of soft targets and enhancing the generalization capabilities of the student model. By default, models are trained for 100 epochs, with the learning rate reduced by a factor of 0.1 at the 40th epoch. We train our models using the Adam optimizer (Kingma and Ba, 2015) with a base learning rate of $2\times 10^{-4},\;\beta_{1}=0.9,\beta_{2}=0.999$ , and a weight decay of $10^{-4}$ . The learning rates for the linear projections, which are used for predicting object query reference points and sampling offsets, are scaled by a factor of 0.1. Runtime evaluations are conducted on an NVIDIA Tesla V100 GPU.

## 5 Discussion

To underscore the merits of our research, we have conducted comprehensive comparisons with existing state-of-the-art (SOTA) weed detection techniques. Our approach exhibits superior performance. Conventional methods typically depend on either purely CNN-based architectures or more intricate and computationally demanding Transformer models. For instance, models such as ViT and Swin Transformer (Liu et al., 2021) achieve comparable performance with CNN-based models on various vision tasks like Image Classification, Object Detection, and Semantic Segmentation on datasets such as ImageNet (Deng et al., 2009), COCO (Lin et al., 2014), and ADE20K (Zhou et al., 2017), respectively. While these models perform well on various tasks, they still face challenges related to high computational costs due to the self-attention mechanism, which scales quadratically with the sequence length. This makes them resource-intensive and challenging to train on standard hardware. These models can be expensive and require large datasets to perform well. Additionally, fine-tuning for specific tasks can be resource-intensive. To produce an automatic weed detection/recognition, In (Zhang, 2023) they created a CNN-Transformer hybrid model effectively captures both local and global features, making it a robust and efficient solution for weed recognition, especially suitable for edge device deployment. Despite this progress, challenges persist, such as the diverse and numerous weed species complicating model training and increasing computing resource demands. Variations in shooting angles and lighting conditions also affect model stability and accuracy (Ghofrani and Mahdian Toroghi, 2022). A novel knowledge distillation technique enhances the small model’s accuracy by transferring knowledge from the large model. Applied to the PlantVillage dataset, this method achieves high accuracy of 97.58% close to the large Xception model’s 99.73%, improving the classification rate of the small model and facilitating early intervention to protect agricultural productivity. In (He et al., 2024), the EDS-YOLOv8 model enhances weed detection by integrating Efficient ViT and RepViT architectures, advanced attention mechanisms (SimAM, EMA, BiFormer), and dynamic snake convolution, resulting in significant improvements in precision, recall, and mAP metrics. This approach aligns with recent advances in weed detection, where hybrid CNN-Transformer architectures have demonstrated remarkable effectiveness. Studies have shown that incorporating attention mechanisms and multiscale feature extraction significantly improves detection accuracy (Xiang et al., 2023), with EM-YOLOv4-Tiny achieving 94.54% mAP for peanut weed detection (Zhang et al., 2022) and SWFormer reaching 76.54% mAP for rapeseed applications (Jiang et al., 2024). Similarly, ConvViT's integration of convolutional and Transformer structures achieved 96.85% accuracy for apple disease identification while maintaining computational efficiency (Li and Li, 2022). The consistent success of attention-enhanced hybrid architectures across different agricultural applications validates the design choices implemented in EDS-YOLOv8. Consequently, The experimental comparison with existing methods demonstrates that our approach effectively addresses several key limitations prevalent in prior studies. First, by employing a lightweight architecture optimized through soft knowledge distillation, our model achieves performance levels comparable to those of larger CNN-based models while significantly reducing training costs. Notably, this strategy obviates the need for extensive training datasets, making the framework more scalable and resource-efficient. Second, the streamlined architecture of our student model ensures seamless deployment on edge devices, a critical requirement for real-time weed detection in agricultural environments. This design not only minimizes computational overhead but also enhances energy efficiency, rendering our solution both practical and sustainable for field applications. Third, the distillation process from a CNN-based teacher model (ResNet-50) confers additional advantages, including the incorporation of beneficial inductive biases and local feature extraction capabilities, while preserving the global receptive field enabled by the attention mechanism. This hybrid learning paradigm allows our model to leverage the strengths of both CNNs and Vision Transformers, resulting in more robust feature representation. Empirical results underscore the efficacy of our method, which surpasses several state-of-the-art (SOTA) approaches across multiple metrics. Specifically, our model achieves a mean average precision (mAP) of 83.47%, along with a precision of 87.21% and recall of 73.93%. Furthermore, it attains an inference speed of 54 FPS making it suitable for real-time applications—while reducing the parameter count of the teacher model by 77.7%, thereby optimizing both performance and computational efficiency. These advancements highlight the potential of our framework to bridge the gap between high accuracy and deployability in precision agriculture, offering a viable solution for resource-constrained environments.

## 6 Conclusion

In this paper, we have investigated weed detection by employing knowledge distillation with an attention mechanism to optimize Vision Transformer (ViT)-based models. Our objective was to develop a lightweight model that can be trained with a relatively small dataset and is cost-effective to deploy, facilitated by the application of soft distillation. To achieve this, we leveraged the capabilities of a pretrained Convolutional Neural Network (CNN) as the teacher model, capitalizing on the benefits of CNNs, such as parameter sharing, inductive biases, and local feature extraction. This knowledge was then transferred to our simplified Vision Transformer through a knowledge distillation technique, augmented with an attention mechanism. This approach significantly enhances the performance of the student model in terms of mean Average Precision (mAP), the number of parameters, and frames per second (FPS). Additionally, being inherently a feature-based method, it can be seamlessly combined with logit-based distillation techniques to further augment the student model’s capabilities. Future research on more precise hyperparameter tuning and data augmentation for our student model is likely to yield significant improvements. Additionally, exploring advanced methodologies such as Grad-CAM guided knowledge distillation could further enhance student model performance by leveraging attention-based feature transfer mechanisms. Furthermore, adopting novel architectures as teacher models could provide richer feature representations and improved knowledge transfer capabilities, thereby benefiting the overall distillation process. Ultimately, the development of efficient computer vision models for weed detection substantially improves weed management practices, fosters sustainable agriculture, and enhances both profitability and environmental sustainability within precision farming.

## Data Availability

The original contributions presented in the study are publicly available. This data can be found here: https://zenodo.org/records/14920303.
